# Physical activity trajectories at older age and all-cause mortality: A cohort study

**DOI:** 10.1371/journal.pone.0280878

**Published:** 2023-01-26

**Authors:** Lamiaa Hassan, Peter Huhndorf, Rafael Mikolajczyk, Alexander Kluttig

**Affiliations:** 1 Institute of Medical Epidemiology, Biostatistics, and Informatics, Medical Faculty of the Martin-Luther-University Halle-Wittenberg, Halle (Saale), Germany; 2 Interdisciplinary Center for Health Sciences, Medical Faculty of the Martin-Luther-University Halle-Wittenberg, Halle (Saale), Germany; University of Tampere, FINLAND

## Abstract

**Background:**

A physically active lifestyle is recognized as a precondition of healthy aging. However, the majority of studies exploring its association with mortality in cohorts of adults used single-time physical activity (PA) estimate, which do not consider its dynamic nature with changes that occur with aging. The aim of the present study is to explore the presence of different PA trajectories in a population-based cohort and their association with mortality.

**Methods:**

We used data of the population-based cohort study CARLA and included 1041 older adults (45–83 years at baseline) with self-reported physical activity at baseline (2002–2006), first follow-up (2007–2010) and second follow-up (2013). Trajectories were identified using growth mixture modelling. Cox proportional hazard models were used to assess the association between trajectories of PA and all-cause mortality during ~6 years since the second follow-up after adjusting for age, sex, lifestyle factors and comorbidities and after correction for classification error. In a sensitivity analysis we weighted the models to account for selection bias during follow-up. As a further sensitivity analysis, we excluded the first year of follow-up to account for reverse causation.

**Results:**

Three PA trajectories (categorized as consistently low, consistently moderate, and high at baseline but strongly decreasing PA across time) were identified, and 121 deaths due to all causes occurred. Compared with participants who had consistently low PA-levels throughout the follow-up period, participants who maintained moderate PA-levels were at a lower risk of all-cause mortality (hazard ratio [HR], 0.49; 95%CI, 0.30–0.70). Participants with high PA-levels at baseline but strongly decreasing PA across time, had similar mortality risk compared to the participants with consistently low PA-levels (hazard ratio [HR], 0.97; 95%CI, 0.50–1.80). The effects were strengthened in the analysis weighted for selection bias.

**Conclusions:**

Our results suggest that, compared to those who had consistently low PA levels, those who maintained a moderate level of PA showed a protective effect in terms of their mortality risk but not those who displayed a decline from high PA levels.

## Introduction

Physical activity (PA) assessed at a single time point is associated with a decrease in all-cause mortality [1]. In a cohort study of 403 681 participants, higher levels of self-reported PA at a single time point were associated with lower all-cause mortality [2]. A meta-analysis of a total of 33 studies with 883372 participants found that PA is associated with a marked decreased in cardiovascular and all-cause mortality in both men and women, even after adjusting for other relevant risk factors [3], A common methodological limitation of using single time point while exploring the association between PA and adverse outcomes in older populations is that it does not account for the dynamic nature of PA behaviors. Additionally, major life changes such as retirement are periods when PA may be subjected to change [4]. It is plausible that prospective trajectories (patterns) of PA levels across time may influence adverse outcomes distinctly as compared with cross-sectional estimates. Previous studies suggest that initiation or maintenance of an active lifestyle can reduce mortality risks [5–8] but the majority of these studies captured change across two time points only. Few studies have examined long-term changes in physical activity and assessed the health impact of various activity trajectories on a population level [9]. Furthermore, conventional methods for defining PA and PA changes are theory driven, which typically involves dividing subjects into cut points either arbitrarily or based on multiples of resting metabolic rate (i.e., inactivity, light, moderate, vigorous activity) [10–13]. This approach, however, relies on the assumption that these patterns truly exist. By contrast, data-driven approaches, such as growth mixture modelling (GMM) or group-based trajectory models, allow the most naturally occurring trajectories to emerge from the data rather than assuming predetermined trajectories [14]. Moreover, using growth mixture modelling or other trajectory modeling, allows researchers to summarize complex patterns of data across the life course instead of using single data points. This type of analysis allows the overall developmental course of the behaviors to be examined [14]. While previous work has explored the association between trajectories of PA and mortality using these data-driven methods, however, these studies, however, were either limited to only male study participants [15, 16], examined only leisure time PA or included populations of a younger age [17]. The association of PA-trajectories and mortality risk in an aging population remains not fully explored. The CARLA study population offers an opportunity to examine the effect of data-driven trajectories based on PA during leisure time and sport time setting on survival in a representative population-based cohort of older adult men and women.

Our aim is to investigate the prospective associations of long-term trajectories of PA on all-cause mortality in an older adults general population. We hypothesized that participants who sustained the highest levels of PA activity in all age groups would have the lowest risk for mortality.

## Material and methods

### Study population

**Fig 1:**
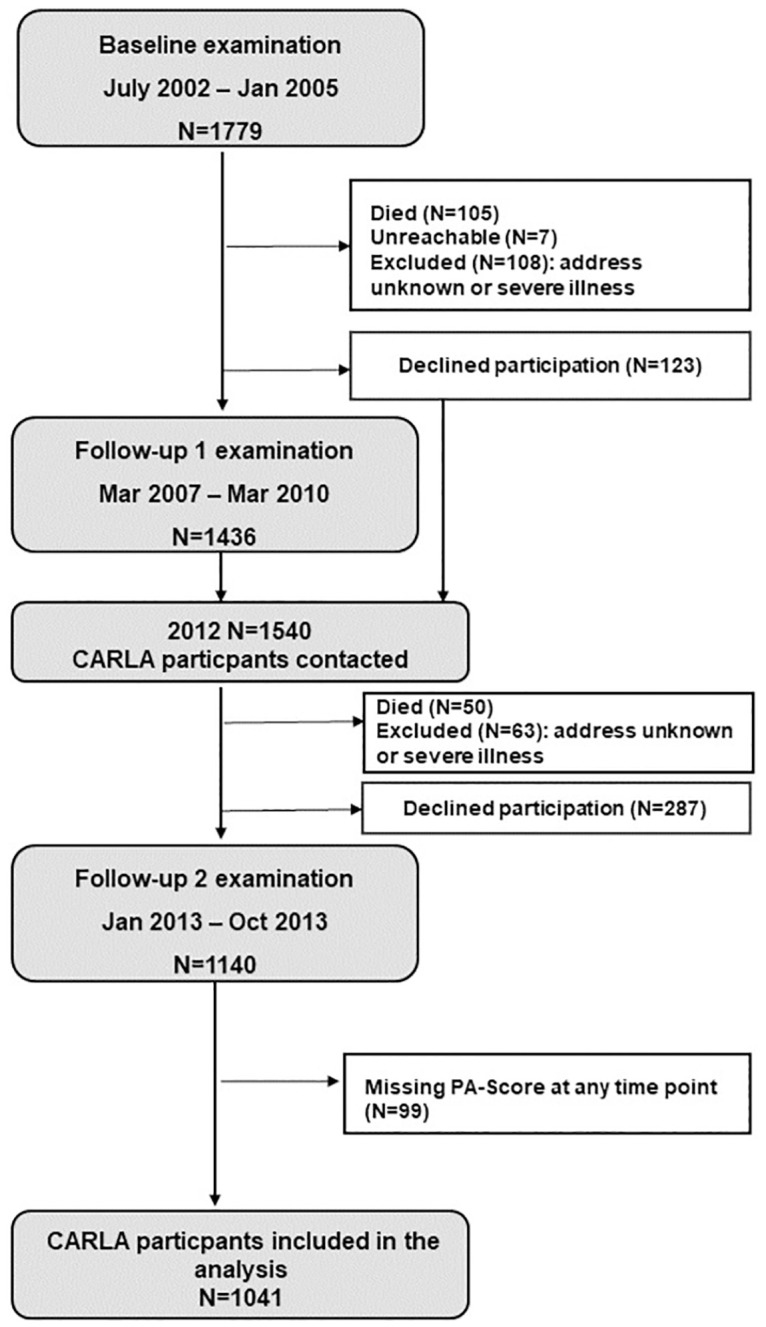
Flowchart of the 1041 CARLA participants included in the analysis.

The Cardiovascular Disease, Living and Ageing in Halle study (CARLA) is a cohort study of a representative sample of the citizens of the city of Halle in eastern Germany with a total of 1,779 participants (age range 45–83 years of age at baseline; 812 women). Details of the study design and methods have been described elsewhere [18–20]. The baseline examination took place between December 2002 and January 2006. After a mean of 4.0 years (standard deviation [SD] = 0.3), 1436 (86%) subjects took part in the first follow-up examination between March 2007 and March 2010. A second follow-up was performed after another 4.8 years (SD = 0.7) between January 2013 and October 2013 with 1136 (77%) participants. For the present study, we included all subjects who took part in the examination and had complete data on PA in all three waves (N = 1041) (58.5%) (Fig 1). 482 (27.1%) subjects were lost due to attrition or had incomplete PA measurements. S1 Table shows the baseline characteristics of the subjects included in the analysis and those who were lost due to attrition or incomplete PA measurements. In general, the group not included in the analysis had a higher mean age of 66.5 years (SD = 10.4), comprised of more females (51.5%), and had lower percentage of current smokers. Moreover, there was a higher percentage of prior cancer, stroke, and myocardial infarction. The original study was approved by the Ethics Committee of the Medical Faculty of the Martin-Luther University Halle-Wittenberg and by the State Data Privacy Commissioner of Saxony-Anhalt and conforms to the principles outlined in the Declaration of Helsinki. All subjects gave written informed consent.

### Questionnaire

Domain specific physical activity was assessed using the well-established Baecke questionnaire [21]. This questionnaire evaluated the level of domain specific physical activity [22, 23]. Briefly, the questionnaire consists of 16 questions in three distinct sections: physical activity during leisure time (LTPA) excluding sport (i.e. one’s own LTPA compared to others of similar age as well as sweating, watching television, walking and cycling during leisure time), sport setting (SPA) (i.e. identification of the sports played followed by questions regarding duration per week and months per year) and work related physical activity (WPA) (i.e. one’s own WPA compared to others of similar age followed by questions regarding sitting, standing, walking, lifting of heavy loads and sweating at work as well as if one is tired after work). Most questions are scored on a five-point Likert scale, ranging from never to always or very often. The three derived indices, LTPA, SPA and WPA, are scored in arbitrary units ranging from 1 to 5. The Baecke questionnaire has been used in a number of studies in various populations and its validity as well as reliability have been thoroughly tested (intraclass correlation from 0.65 to 0.92 and a Pearson correlation with doubly labeled water of 0.54 to 0.69) [21, 22, 24–26]. Since 60% of the participants were already retired at baseline and no relationship was found between WPA and mortality in a previous analysis [6], only LTPA and SPA were used for this analysis.

### PA total score

The total PA score per individual was formed from the sum of the LTPA and the SPA index, each scored in arbitrary units ranging from 1 to 5. The total score range starts at a value of 2 (no reported PA) and goes up to 10 (maximum reported PA).

### Mortality

For all subjects included in our study, mortality was recorded from the date of the second follow-up examination (2013) until June 2019 (Fig 1). All-cause mortality was determined after a median follow-up of 6.1 years (25th and 75th percentile: 5.9–6.3). Information on the vital status of the participants was obtained via a query at the residents’ registration office. For deceased participants, we requested a copy of the official death certificate from the local health authority. More information on mortality registration is found elsewhere [20].

### Covariates

The standardized, computer-assisted interview collected information regarding sociodemographic variables like educational level attained, lifestyle factors like smoking and alcohol consumption. Subjects were asking if they are current or past smokers or if they have never smoked and the variable was accordingly coded. Alcohol consumption was coded as in gram/day consumption. Self-reported information about physician-diagnosed incident cancer, diabetes, and cardiovascular events (myocardial infarction, stroke) were collected in the interview. Weight and height were measured using the SECA-107 digital scale and SECA-220 height measuring system [18], respectively, and used to calculate the BMI. Information on the use of statin medication during the seven days preceding the examination was collected by the study nurse using the computer-based IDOM program [27]. More detailed description of the collection and assessment of the covariable is found elsewhere [20].

### Statistical analysis

General descriptive statistics were calculated for the baseline characteristics of the population. Continuous variables were displayed as means and standard deviations. Categorical variables were displayed as numbers and percentages.

#### PA trajectories

To determine trajectories of physical activity, GMM was conducted using the R package “lcmm”.

The growth function applied fits latent class linear mixed models (LCLMM) also known as GMM or heterogeneous linear mixed models. The indicator variable was the first, second and third measurement of the total score. Age, sex, lifestyle risk factors and comorbidities at the baseline examination were included as active covariates in model estimation, meaning that posterior probability for membership for each trajectory could vary as a function of these covariables. Models with 2–5 trajectory groups were run using 200 random sets and 100 iterations and compared using goodness-of-fit criteria. The fit of the model was determined using the following criteria [28–30]: 1) Probability and proportion assigned, 2) Average posterior probability > .7, 3) Odds of correct classification > 5, 4) % of iterations converging on the same solution, 5) Number of cluster model selected from those with satisfactory overall fit, 6) the minimum values of the goodness of fit measures Bayes Information Criterion (BIC), Akaike’s Information Criterion (AIC) as indicators of the optimal number of classes, 7) the degree to which the trajectory classes identified captured distinct and meaningful patterns in the data, and 8) the quality of the model based on entropy R [31]. Average posterior probabilities of membership were computed for each group to estimate the reliability of the classification. Individual posterior probability of membership for a subject represents his/her probability of belonging to the group he/she is assigned to by previous grouping based on his/her individual features. Trajectory average posterior probability of membership represents its internal consistency, with higher values indicating better classification quality. Descriptive statistics for demographic, lifestyle and CVD risk factors are presented according to trajectory groups.

As recommended by Hickson et al. [32], Spaghetti plots of the trajectories were drawn to provide further diagnostic information about model fitness by detecting through visual inspection whether individual patients’ PA patterns within a trajectory group are homogeneous.

#### Survival analysis

Using Cox proportional hazard regression, we estimated the hazard ratios (HR) and 95% confidence intervals (CI) of all-cause mortality risk in regard to the three PA-Groups with Group 1 as the group with lowest activity levels over time as the reference group.

We investigated the mortality risk in four models. Model 1 included no further covariates other than age and sex; in further analyses, we included covariates in a stepwise manner. Covariates were first grouped in two separate groups to differentiate the underlying effects of the health-related lifestyle risk factors versus the effects of the chronic diseases and their indicators (Model 2 vs. Model 3). Both groups of covariates were then included in Model 4. In all models, we included weights for the individual posterior probability of membership of the trajectory to account for possible classification errors.

The assumption of proportionality of hazards was confirmed by assessing the Schoenfeld residuals for Model 4 using the cox.zph() function of the ‘survival’ package in R. There were no significant p-values found indicating that there are no time dependent coefficients in the model. We plotted the scaled Schoenfeld residuals for Model 1 which showed no non-zero slopes (S1 Fig).

As our analysis comprised of 58.5% of the original study population, we also performed a weighted cox regression analysis by weighing each subject by the inverse probability of having participated in the first and second follow-up. Weights were based on baseline characteristics affecting participation: age, sex, educational level, and PA level at baseline. The weights were calculated using the R package “ipw”.

To account for possible reverse causation, we performed a sensitivity analysis, in which we considered 2013 a ‘wash-out’ period and censored those who died in 2013. As a subgroup analysis, we also investigated the mortality risk of the subgroup formed based on the visual inspection as an additional analysis to check the robustness of the main analysis estimates.

## Results

### Baseline characteristics

Out of 1041 subjects, 121 (11.6%) died during a mean duration of follow-up time of 6.2 years from the second follow-up examination. At baseline, those who deceased were older and had lower PA score (5.7 [±1.1] vs. 5.5 [±1]). The deceased group comprised of more men with a higher proportion of smokers and ex-smokers. Furthermore, there was a higher percentage of prior cancer, myocardial infarction, and use of statin medication.

### PA-Trajectories characteristics

**Fig 2:**
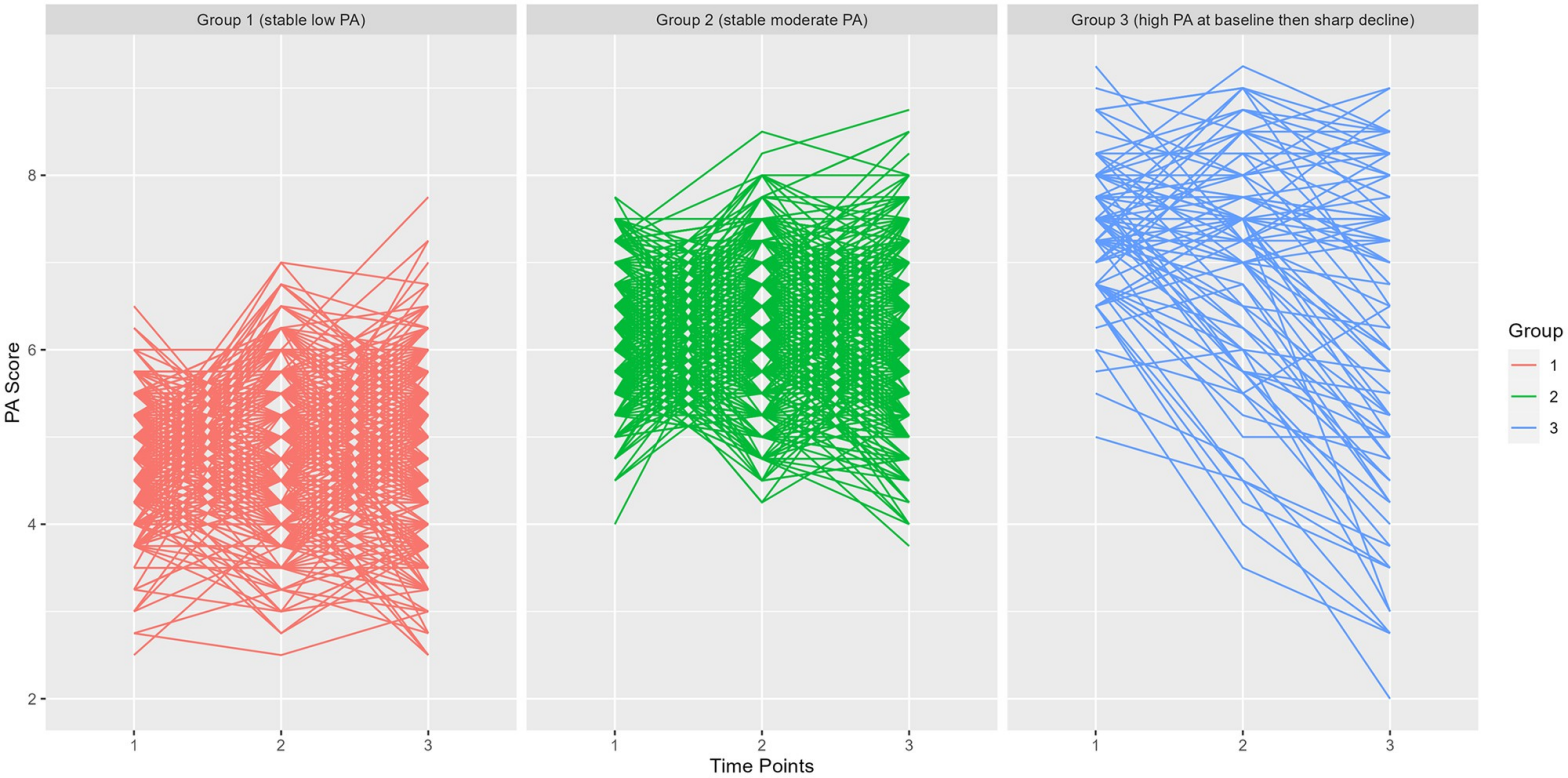
Regression lines of the participants in each trajectory based on the Physical Activity (PA) measurements of 1041 CARLA participants at 3 time points.

**Fig 3:**
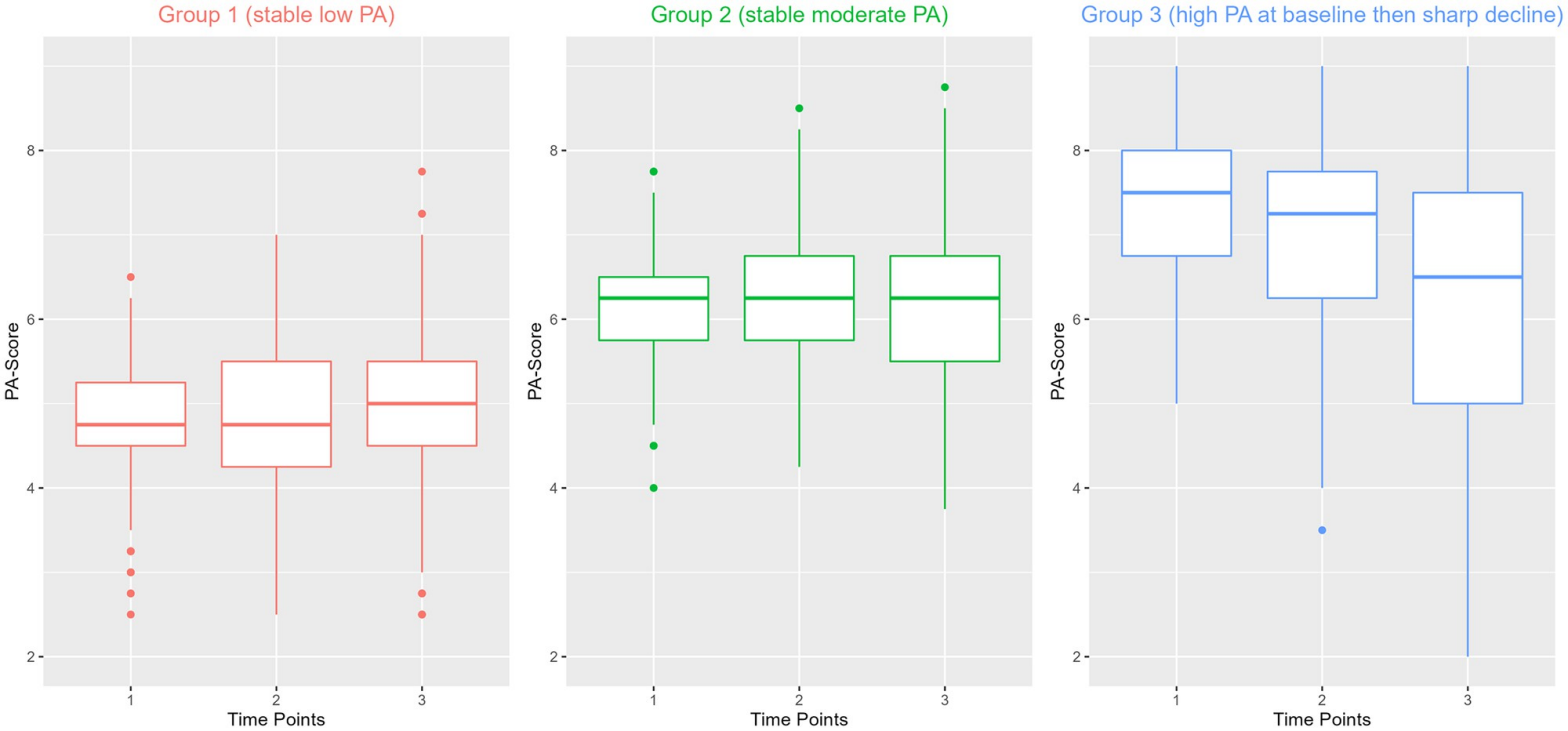
Boxplot showing the mean PA-Score across three time points of the 1041 CARLA participants according to their PA-Trajectories.

The 3-group GMM model was chosen based on having better Goodness-of-fit statistics shown by the Log-likelihood (-321.06), AIC (676.13) and BIC (717.65) values and a higher entropy value (82.23%) than values of the models with 2, 4 and 5 groups (S2 Table). The GMM determined three PA-Trajectories shown in Figs 2 and 3. Group 1 had a stable relatively low PA-Score across the three time points while Group 2 had a relatively stable moderate PA-Score along the 3 time points. Group 3 started at baseline with a high PA-Score and showed a sharp decline in PA-Score in the second and third time point (Figs 2 and 3).

Table 1 shows the baseline characteristics of the PA trajectory groups. Group 1 and 2 each differ from Group 3 by a relatively lower educational status. In Group 1 and 3, 14% of the participants, 65 and 12 participants respectively, died during the follow-up period while in Group 2 44 (8.9%) died.

**Table 1:** Characteristics of PA-Trajectories of 1,041 CARLA subjects at the second FU examination.

		Group 1 (Stable low PA) N = 458 (44%)	Group 2 (Stable moderate PA) N = 497 (47.7%)	Group 3 (High PA at baseline then sharp decline) N = 86 (8.3%)
**Age (years), Mean (SD)**		69.2 (9.2)	70.3 (8.7)	70.3 (9.0)
**Sex (Female)**		215 (46.9%)	231 (46.5%)	37 (43%)
**BMI (kg/ m** ^**2**^ **), Mean (SD)**		28.7 (4.5)	29.1 (4.8)	29.7 (5.8)
**Alcohol (g/d), Mean (SD)**		10.5 (17.3)	9.9 (14)	12.1 (17.9)
**Total score at baseline, Mean (SD)**		4.8 (0.6)	6.2 (0.7)	7.4 (0.8)
**Total score at FU1, Mean (SD)**		4.9 (0.7)	6.2 (0.8)	7.1 (1.3)
**Total score at FU2, Mean (SD)**		4.9 (0.8)	6.1 (0.9)	6.2 (1.7)
**Sport time score at baseline, Mean (SD)**		1.9 (0.4)	2.7 (0.6)	3.5 (0.6)
**Sport time score at FU1, Mean (SD)**		2.0 (0.6)	2.9 (0.7)	3.4 (0.9)
**Sport time score at FU2, Mean (SD)**		2.1 (0.6)	2.9 (0.7)	2.9 (1.1)
**Leisure time score at baseline, Mean (SD)**		2.9 (0.5)	3.4 (0.5)	3.9 (0.5)
**Leisure time score at FU1, Mean (SD)**		2.8 (0.6)	3.4 (0.5)	3.7 (0.7)
**Leisure time score at FU2, Mean (SD)**		2.8 (0.6)	3.3 (0.6)	3.3 (0.9)
**Smoking**	**Never**	215 (46.9%)	230 (46.4%)	38 (44.2%)
	**Ex-Smokers**	165 (36.0%)	214 (43.1%)	36 (41.9%)
	**Current**	68 (14.8%)	42 (8.5%)	10 (11.6%)
	**Occasional**	10 (2.2%)	10 (2.0%)	2 (2.3%)
**Education Level**	**Low**	30 (6.6%)	21 (4.2%)	2 (2.3%)
	**Intermediate**	309 (67.5%)	279 (56.1%)	49 (57.0%)
	**High**	119 (26.0%)	196 (39.4%)	35 (40.7%)
**Myocardial Infarction**		39 (8.6%)	38 (7.7%)	8 (9.3%)
**Stroke**		22 (4.9%)	20 (4.0%)	3 (3.5%)
**Cancer**		68 (14.9%)	82 (16.7%)	6 (7.0%)
**Lipids/ Statins Medication**		138 (30.1%)	124 (24.9%)	21 (24.4%)
**Dead in the follow up until June 2019**		65 (14.2%)	44 (8.9%)	12 (14.0%)

**Fig 4:**
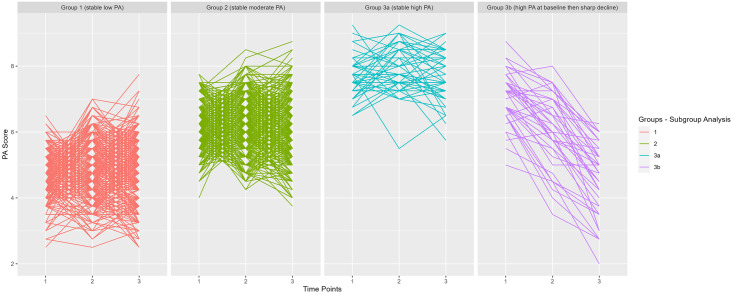
Regression lines of the participants in each trajectory based on the Physical Activity (PA) measurements of 1041 CARLA participants at 3 time points in a subgroup analysis where Group 3 is divided into 2 Groups: 3a and 3b.

Visual inspection of the Spaghetti plot suggested that Group 3 is a heterogeneous group (Fig 2). We ran the GMM analysis on this group only. There were two distinct subgroups within Group 3: one with participants who kept high levels of PA throughout from baseline till the second Follow Up and the second one had participants with an initially high PA level and but then experienced a sharp decline (Fig 4).

### Effect of PA-Trajectories on overall mortality

Compared to Group 1 with the lowest PA-trajectory, Group 2 that showed moderate stable PA-score had a lower mortality risk (HR: 0.52 95% CI: 0.4–0.8) after adjusting for age and sex and weighting for classification error (Table 2). Adjustments to the lifestyle factors of smoking and alcohol, BMI, and educational attainment (Model 2) strengthened the effect. The effect was strengthened after adjusting for myocardial infarction, cancer, stroke, and lipid-lowering medication use (as reported or measured at baseline) in the final Model 4 (HR: 0.49 95% CI: 0.3–0.7). Group 3, which had a high PA-Score at baseline and showed a decline in time point 2 and 3, showed no difference in the mortality risk compared Group 1 with an HR of 0.98 (95% CI: 0.5–1.8) in Model 1 and the effect remained unchanged in Model 4 after adjusting for lifestyle factors and comorbidities (HR: 0.97 95% CI: 0.5–1.8) (Table 2). The analysis weighted for selection bias during follow-up shows an overall strengthening of the effect for Group 2 (Model 4: HR: 0.47 CI: 0.3–0.7) and 3 (Model 4: HR: 0.83 CI: 0.5–1.4). The sensitivity analysis where those who died 2013 were censored, also showed a constant strengthening of the effect for Group 2 and Group 3 across all Models with an HR of 0.46 (95% CI: 0.3–0.7) for Group 2 and an HR of 0.76 (95% CI: 0.4–1.6) for Group 3 (S3 Table).

**Table 2:** Unweighted and weighted estimates of all-cause mortality risk of the groups according to activity category using Cox regression models.

	Activity category	Group Size (N)	Adjusted HR (95% CI) Model 1	Adjusted HR (95% CI) Model 2	Adjusted HR (95% CI) Model 3	Adjusted HR (95% CI) Model 4
**Unweighted analysis**	**Group 1 (Stable low PA) (Reference Group)**	**N = 458**	1.00	1.00	1.00	1.00
	**Group 2 (Stable moderate PA)**	**N = 497**	0.52 (0.4–0.8)	0.48 (0.3–0.7)	0.54 (0.4–0.8)	0.49 (0.3–0.7)
	**Group 3 (High PA at baseline then sharp decline)**	**N = 86**	0.98 (0.5–1.8)	1.03 (0.5–1.9)	0.93 (0.5–1.8)	0.97 (0.5–1.8)
**Weighted analysis**	**Group 1 (Stable low PA) (Reference Group)**	**N = 458**	1.00	1.00	1.00	1.00
	**Group 2 (Stable moderate PA)**	**N = 497**	0.52 (0.4–0.8)	0.48 (0.3–0.7)	0.56 (0.4–0.8)	0.47 (0.3–0.7)
	**Group 3 (High PA at baseline then sharp decline)**	**N = 86**	0.90 (0.5–1.6)	0.93 (0.5–1.6)	0.85 (0.5–1.6)	0.83 (0.5–1.4)

PA: physical activity. Model 1 is adjusted for age, sex Model 2: Model 1+ smoking, alcohol, BMI, years of education Model 3: Model 1 + myocardial infarction, stroke, cancer, use of lipid-lowering drugs Model 4: Model 1+2+3. All models have been weighted for the individual posterior probability to account for classification errors.

In the subgroup analysis where Group 3 was divided into Group 3a (consistently high PA, N = 46) and 3b (initial high and strongly declining, N = 40), both groups, the former one with an HR of 0.79 (0.4–2.2) and the latter (HR: 0.97 95%CI: 0.6–2.2) showed no difference concerning the mortality risk compared to Group 1 (Table 3). Both effect estimates had wide CI due to the relatively small group size.

**Table 3:** All-cause mortality risk of the groups according to activity category using Cox regression models in a subgroup analysis.

Activity category	Group Size (N)	Adjusted HR (95% CI) Model 1	Adjusted HR (95% CI) Model 2	Adjusted HR (95% CI) Model 3	Adjusted HR (95% CI) Model 4
**Group 1 (Stable low PA) (Reference Group)**	**N = 458**	1.00	1.00	1.00	1.00
**Group 2 (Stable moderate PA)**	**N = 497**	0.56 (0.4–0.8)	0.52 (0.3–0.8)	0.54 (0.4–0.8)	0.50 (0.3–0.8)
**Group 3a (Stable high PA)**	**N = 46**	0.74 (0.3–2.1)	0.79 (0.3–2.2)	0.79 (0.3–2.2)	0.79 (0.4–2.2)
**Group 3b (High PA at baseline then sharp decline)**	**N = 40**	1.12 (0.5–2.3)	0.98 (0.4–2.2)	1.10 (0.5–2.3)	0.97 (0.6–2.2)

PA: physical activity. Model 1 is adjusted for age, sex Model 2: Model 1+ smoking, alcohol, BMI, years of education Model 3: Model 1 + myocardial infarction, stroke, cancer, use of lipid-lowering drugs Model 4: Model 1+2+3. All models have been adjusted for the individual posterior probability to account for classification errors.

## Discussion

In an older adults general population cohort, with self-reported PA measured at baseline and at two follow-ups with four years each in between, we confirmed previous findings regarding association of maintaining moderate levels of PA levels with a reduced risk of mortality, independently of important confounding factors. Furthermore, we found that participants who displayed a decline from high PA levels had similar mortality to those who maintained low PA levels. In the weighted analysis the results remained consistent and showed a strengthened effect. In a subgroup analysis, we found indications that continuous high PA might also have protective effects. However, the effect was smaller than for continuous moderate PA and imprecisely estimated because of the small number of participants in the subgroups.

Our findings concerning PA trajectories and mortality cannot not be directly compared to the previous literature due to the specific subgroups identified using the group-based trajectory models and other methodological differences. We note, however, that the results in this paper are largely consistent with previous studies, which used similar data-driven methods. In 3231 British adult men between the age of 40 and 59 years, Aggio et al. found that belonging to the group with light/stable (HR 0.83, 95% CI 0.74 to 0.94) or moderate/increasing (HR 0.76, 95% CI 0.66 to 0.88) PA levels was associated with a 17% and 24%, respectively, lower risk of all-cause mortality compared to the low/decreasing group [15]. Laddu et al. assessed PA of 3767 men aged ≥65 years of age at up to four time points, evaluating all-cause mortality [16]. Their analysis identified three discrete PA patters all with declining PA where the moderate (HR = 0.78; 95% confidence interval [CI]: 0.70 to 0.88) and high-activity (HR = 0.69, 95% CI: 0.57 to 0.83) declining groups were associated with a lower all-cause mortality risk compared to the low-activity declining men. Our results show a stronger effect (HR = 0.50, 95% CI: (0.3–0.7)) for the group with consistent moderate PA levels compared to the low-PA levels group. However, these previous two studies included only men. Sanchez-Sanchez et al. sought to explore associations between PA trajectories and mortality in a Spanish older adult cohort of 1679 subjects (mean age 74.94 ± 5.32 years; 59% women) using a growth mixture model along almost 6 years follow-up [33]. They found that compared to the group with the high PA-consistent group, belonging to the low PA-decreasing group had higher mortality risk (HR = 1.68; 95%CI = 1.21 to 2.31) which is an effect similar in the magnitude of the estimates of our results. In a study by Saint-Maurice of 315 059 participants, ten trajectories of leisure-time PA were identified and they found that compared with participants who were consistently inactive, maintaining higher amounts of LTPA was associated with lower all-cause (HR = 0.64; 95%CI, 0.60–0.68) which is consistent with our results [17].

When comparing with studies which did not use data-driven methods in identifying the trajectories we found some differences. In a study by Mok et al. of 14 599 men and women aged 40–79 years followed for median of 12.5 years, increasing physical activity was found to be beneficial compared to consistent inactivity irrespective of the baseline PA value [34]. However, using GMM, we did not identify groups of older adults that made substantial positive changes in PA, highlighting that older adults who are shifting from low or modest to even modest or high levels of PA, respectively, are rare. Instead, the GMM trajectories suggest that previous PA commands PA in later age stages. The analysis, however, identified a relatively small heterogeneous group with appeared to have an initial high PA level that experienced a rapid decline. This group, however, did not differ in regards in their mortality risk from the group with low consistent PA levels, which suggests that maintaining consistently moderate levels of PA levels at higher age is optimal, and persons with high PA levels are at risk of activity declaim with subsequently reduced protective effect. In our subgroup analysis, it was shown that the protective effect for the Group 3a, which had consistently high PA-level compared to the group with consistently low PA-levels, was weaker than the effect for the group with consistently moderate PA-levels and because it was imprecisely estimated due to the small group size, it should be interpreted with caution.

Regarding the GMM algorithm classification quality, the identified trajectory groups displayed mean posterior probabilities of membership that were close to 0.8. As values of 0.7–0.8 are thought to be indicative of an aggrupation that sufficiently distinguishes between individuals with different patterns of change in a behavior over time [35], we conclude that our classification is consistent. In addition, our analysis is adjusted for individual posterior probability to account for possible classification errors. Individual participant PA patterns from spaghetti plots provided additional diagnostic information about trajectory models beyond standard model-fit assessments to determine if group-average PA estimates represent homogeneous patterns of PA, which showed homogeneous patters for Group 1 and 2, but not for Group 3.

### Limitation and strength

The strength of the study is the original representative sample of the elderly general population. Weighting was used to try to take into account the selection that took place in the follow-up period. However, this cannot fully guarantee the representativeness of the sample over the entire follow-up period, as weighting can only be performed for observed variables.

Also, the use of the same PA questionnaire at each wave and the inclusion of a range of cofounders. The use of GMM approach permits the most naturally arising trajectories to be found, delivering more accurate estimates of PA over time.

However, this study also has limitations: Self-reported PA may still be subject to recall and social desirability bias. While we control for a range of confounding factors, residual confounding may be present. Unmeasured health issues may lead to decreased mobility and so the risk of reverse causation persists. Furthermore, the GMM methodology used finds dominant patterns of PA over time, but it does not preclude individual variability in PA patterns and may lead to over-grouping with significant variation within PA trajectories. In addition, our sample had the minimum time points allowed for the GMM analysis, which is 3 time points, having more time points probably will lead to more precise identification of the different trajectories. Furthermore, we assessed only PA during an 8-year period in older adulthood; we do not have any information about PA at younger age. Finally, we could not study separate age cohorts due to limited sample size.

## Conclusion

Compared to consistently low PA-levels, consistently moderate PA-levels are associated with lower mortality over a long-time period in the general population, rather than high PA-levels that decline over time. These findings suggest that maintaining moderate PA levels in late adulthood is optimal in terms of reduced mortality.

## Supporting information

S1 Table Suppl baseline characteristics of 1,041 CARLA subjects included in the analysis and 482 CARLA subjects lost to attrition or excluded from the analysis. (DOCX)

S2 Table Model fit statistics for growth mixture models (GMM) estimated using ‘lcmm’ package in R. (DOCX)

S3 Table All-cause mortality risk of the groups according to activity category using Cox regression models censoring those who died in 2013. (DOCX)

S1 Fig Plotted scaled Schoenfeld residuals for Model 1 for group, age, and sex. (TIF)

## Abbreviations

AIC: Akaike’s Information Criterion
BIC: Bayes Information Criterion
CI: Confidence Interval
GMM: Growth mixture modelling
HR: Hazard Ratio
LTPA: leisure time physical activity
PA: Physical activity
SPA: Sport setting physical activity
WPA: Work related physical activity
