# Supplementary material for: Physical activity trajectories at older age and all-cause mortality: A cohort study
Source: PLoS One. 2023 Jan 26;18(1):e0280878. doi: 10.1371/journal.pone.0280878 (PMC9879516; doi:10.1371/journal.pone.0280878)
Supplement: S1 Table — (DOCX) [file pone.0280878.s001.docx]

**S1 Table. Suppl Baseline characteristics of 1,041 CARLA subjects included in the analysis and 482 CARLA subjects lost to attrition or excluded from the analysis**

|  | | **1041 (68.4%) subjects included in the analysis** | **482 (31.6%) Subjects lost to attrition/ left out** |
| --- | --- | --- | --- |
| **Age (years), Mean (SD)** | | 57.3 (6.1) | 66.5 (10.4) |
| **Sex (Female)** | | 458 (44.0%) | 248 (51.5%) |
| **BMI (kg/ m^2^), Mean (SD)** | | 28.4 (5.1) | 28.8 (5.0) |
| **Alcohol (g/d), Mean (SD)** | | 13.4 (18.0) | 9.8 (16.2) |
| **Total score at baseline, Mean (SD)** | | 5.6 (1.1) | 5.4 (1.0) |
| **Sport time score at baseline, Mean (SD)** | | 2.4 (0.7) | 2.3 (0.7) |
| **Leisure time score at baseline, Mean (SD)** | | 3.2 (0.6) | 3.1 (0.6) |
| **Smoking** | **Never** | 430 (41.3%) | 230 (47.7%) |
|  | **Ex-Smokers** | 333 (32.0%) | 152 (31.5%) |
|  | **Current** | 278 (26.7%) | 100 (20.7%) |
| **Education Level** | **Low** | 63 (6.1%) | 67 (13.9%) |
|  | **Intermediate** | 668 (64.2%) | 338 (70.1%) |
|  | **High** | 310 (29.8%) | 77 (16.0%) |
| **Myocardial Infarction** | | 37 (3.6%) | 27 (5.6%) |
| **Stroke** | | 19 (1.8%) | 25 (5.2%) |
| **Cancer** | | 50 (4.8%) | 39 (8.1%) |
| **Lipids/ Statins Medication** | | 91 (18.9%) | 118 (11.3%) |
