# Supplementary material for: Physical activity trajectories at older age and all-cause mortality: A cohort study
Source: PLoS One. 2023 Jan 26;18(1):e0280878. doi: 10.1371/journal.pone.0280878 (PMC9879516; doi:10.1371/journal.pone.0280878)
Supplement: S2 Table — (DOCX) [file pone.0280878.s002.docx]

**S2 Table. Model fit statistics for growth mixture models (GMM) estimated using ‘lcmm’ package in R**

| **Number of groups** | **Log-likelihood** | **AIC** | **BIC** | **Entropy** | **Group-sizes** |
| --- | --- | --- | --- | --- | --- |
| 2-groups | -333.6944 | 693.3887 | 725.1432 | 0.7828818 | 54.1,45.9 |
| **3-groups** | **-321.0635** | **676.1270** | **717.6521** | **0.822313** | **44.0,8.3,47.7** |
| 4-groups | -4063.1048 | 8168.2096 | 8272.1163 | 0.5071786 | 46,45,8,1 |
| 5-groups | -4058.3320 | 8166.6640 | 8290.3624 | 0.5936339 | 28,27,11,33,1 |

AIC: Akaike’s Information Criterion. BIC: Bayes Information Criterion

The lines in bold font represent the models with the best fit according to the criteria
