# Supplementary material for: Physical activity trajectories at older age and all-cause mortality: A cohort study
Source: PLoS One. 2023 Jan 26;18(1):e0280878. doi: 10.1371/journal.pone.0280878 (PMC9879516; doi:10.1371/journal.pone.0280878)
Supplement: S3 Table — (DOCX) [file pone.0280878.s003.docx]

**S3 Table. All-cause mortality risk of the Groups according to activity category using Cox regression models censoring those who died in 2013**

| **Activity category** | **Group Size (N)** | **Adjusted HR**  **(95% CI)**  **Model 1** | **Adjusted HR**  **(95% CI)**  **Model 2** | **Adjusted HR**  **(95% CI)**  **Model 3** | **Adjusted HR**  **(95% CI)**  **Model 4** |
| --- | --- | --- | --- | --- | --- |
| **Group 1**  **(Stable low PA)**  **(Reference Group)** | **N=458** | 1.00 | 1.00 | 1.00 | 1.00 |
| **Group 2**  **(Stable moderate PA)** | **N=497** | 0.52 (0.4–0.8) | 0.46 (0.3–0.7) | 0.54 (0.4–0.8) | 0.46 (0.3–0.7) |
| **Group 3**  **(High PA at baseline then sharp decline)** | **N=86** | 0.84 (0.4–1.6) | 0.84 (0.4–1.7) | 0.76 (0.4–1.5) | 0.76 (0.4–1.6) |

PA: physical activity. Model 1 is adjusted for age, sex Model 2: Model 1+ smoking, alcohol, BMI, years of education Model 3: Model 1 + myocardial infarction, stroke, cancer, use of lipid-lowering drugs Model 4: Model 1+2+3
