# Supplementary figures and images for: Physical activity trajectories at older age and all-cause mortality: A cohort study
Source: PLoS One. 2023 Jan 26;18(1):e0280878. doi: 10.1371/journal.pone.0280878 (PMC9879516; doi:10.1371/journal.pone.0280878)

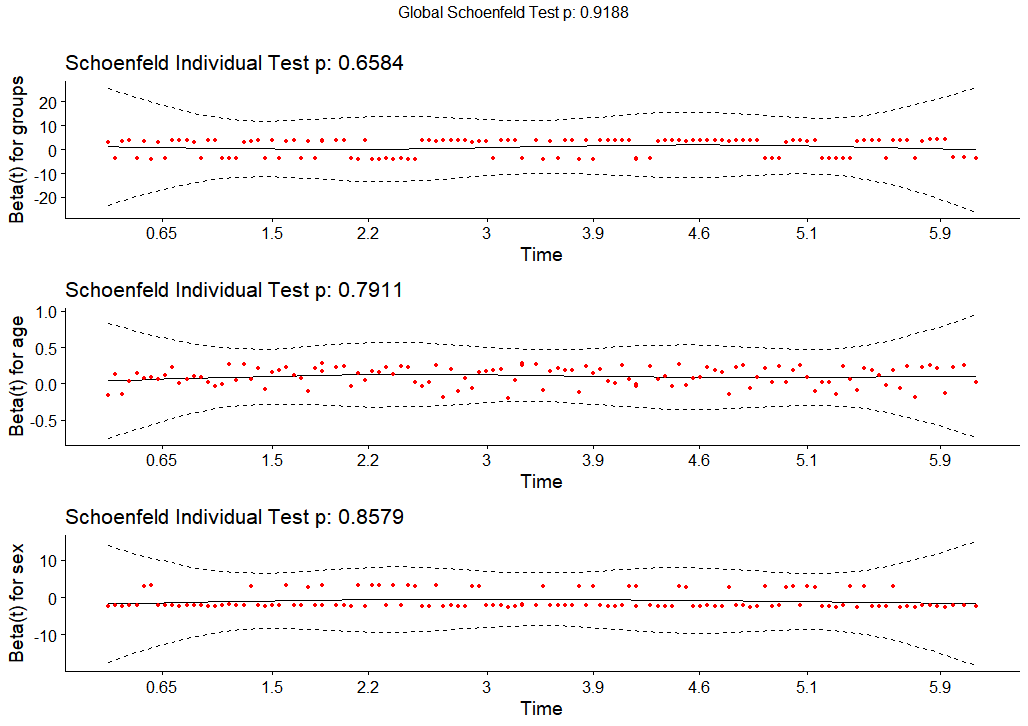

Supplement: S1 Fig — (TIF) [file pone.0280878.s004.tif]
